# Tensile, compressive, and fatigue strength of a quasi-isotropic carbon fiber reinforced plastic laminate with a punched hole

**DOI:** 10.1016/j.heliyon.2020.e05690

**Published:** 2020-12-10

**Authors:** Masahito Ueda, Vu Manh Cuong, Atsushi Yamanaka, Kazuhiro Sakata

**Affiliations:** a Department of Mechanical Engineering, College of Science and Technology, Nihon University, 1-8-14 Kanda-surugadai, Chiyoda, Tokyo, 101-8308, Japan; b Department of Mechanical Engineering, College of Industrial Technology, Nihon University, 1-2-1 Izumicho, Narashino, Chiba, 275-8575, Japan

**Keywords:** Materials science, Mechanical engineering, Automotive engineering, Machining, Materials processing, Polymer-matrix composites, Failure, Joints

## Abstract

The mechanical properties of a quasi-isotropic carbon fiber reinforced plastic (CFRP) laminate with a punched hole were investigated. During the punching process, pressure was applied to the laminate through the upper and lower blank holders of a punching machine; the clearance between the punch and blank holders was set to be small to suppress damage in the CFRP laminates. Due to the dragging of plies encountered during punching, the surface of the punched hole was relatively uneven as compared to that of the drilled hole. However, the effect of the uneven surface created during the punching process was not as significant on the tensile and compressive strength of the open hole as compared to the manufacturing damages generated by drilling processes. The stress–number of cycles to failure curves for the open-hole tension–tension fatigue tests also showed comparable results between the punched- and drilled-hole specimens. These results indicate that there were no significant differences in the mechanical properties of CFRP laminates with a punched hole, and thus present the possibility of a highly productive hole-making process using the punching method.

## 1 Introduction

In hole making, prior mechanical fastening is required. A mechanical fastener such as a bolt or rivet is typically used in combination with adhesive bonding for joining carbon fiber reinforced plastic (CFRP) laminates [1, 2, 3]. Hole making in CFRP laminates can be done using processes such as drilling, abrasive water jet cutting, electro-discharge machining, and laser beam-cutting [4]. However, owing to the processing time and manufacturing cost involved in these processes, the instant hole-making technique is preferred for the mass production of CFRP structures.

Punching is a highly productive and cost-efficient technique to make holes in metallic sheets. However, damage (e.g., delamination and splitting) is easily occurred in CFRP laminates at the point of punching [5, 6, 7]. Therefore, the punching is not a common method in industry to make holes on CFRP laminates.

Punching on CFRP laminates have been studying to improve the quality of the hole. Chan et al. performed punching using a dual-stage puncher to reduce the punching load when a small clearance between the punch and the die was selected [8]. Nakamura et al. and Ueshiba et al. studied the effect of punch shape, clearance, and process temperature on the quality of the hole [9, 10]. Klocke et al. changed the cutting clearance, blank holder pressure, and cutting-edge radius to study the dimensional accuracy of the punched holes [11]. Ho and Yanagimoto studied the effect of ply thickness of CFRP laminates on the punching load [12]. Yashiro and Ogi performed out-of-plane shear tests of CFRP laminates, and they showed that delamination was generated due to the high shear stress required to cut the reinforcing fibers [13]. Duan et al. used the edged punch, flat punch, and hollow punch, and the edge punch showed higher machining accuracy for CFRP laminates [14].

Lambiase et al. conducted mechanical tests to investigate the difference between drilled- and punched-hole materials using a plain-woven glass fiber reinforced epoxy composite [15]. The open hole tensile test with a central hole showed a negligible difference between the drilled-hole and punched-hole specimens with a punch-die clearance of 0.1 mm. However, high-performance CFRP laminates are more sensitive to the out-of-plane impact [6, 7, 16, 17, 18]. Therefore, Abdullah et al. applied drilling after punching to improve the quality of the punched hole on a CFRP laminate [19]. The pin-loading tests of the CFRP laminates showed almost similar bearing strength between the drilling and the combined technique. However, the post-processing after punching needs to be omitted to reduce the cost of manufacturing. To expand the range of applications, punching process needs to be studied further on the CFRP laminates and CFRP/Metal laminates [12, 20].

In this study, two types of open-hole CFRP laminates processed by punching and drilling were prepared and tested. The CFRP laminates were fabricated from unidirectional prepreg sheets, which have been used for high-performance structural applications. Punching on the CFRP laminate was performed minimizing the clearance between the diameters of the punch tool and the blank holder. The open-hole tension (OHT), open-hole compression (OHC), and tension-tension fatigue tests of the quasi-isotropic CFRP laminates were performed to investigate the influence of punching on their mechanical properties. The applicability of the punching technique as a highly productive hole-making process for structural CFRP laminates was investigated in this study.

## 2 Materials and methods

### 2.1 CFRP laminates

Quasi-isotropic CFRP laminates were made using a unidirectional prepreg sheet (T800S/#2592, Toray Industries) of 0.1 mm thickness and 65% fiber volume fraction. The stacking sequence of the laminates was [-45/90/45/0]_2s_, and the total thickness was 1.6 mm. It was cured at 130 °C for 90 min in an oven under vacuum. After curing the laminates, they were cut into rectangular shapes using a cutting machine with diamond grits for mechanical testing.

### 2.2 Specimen preparation

**Figure 1:**
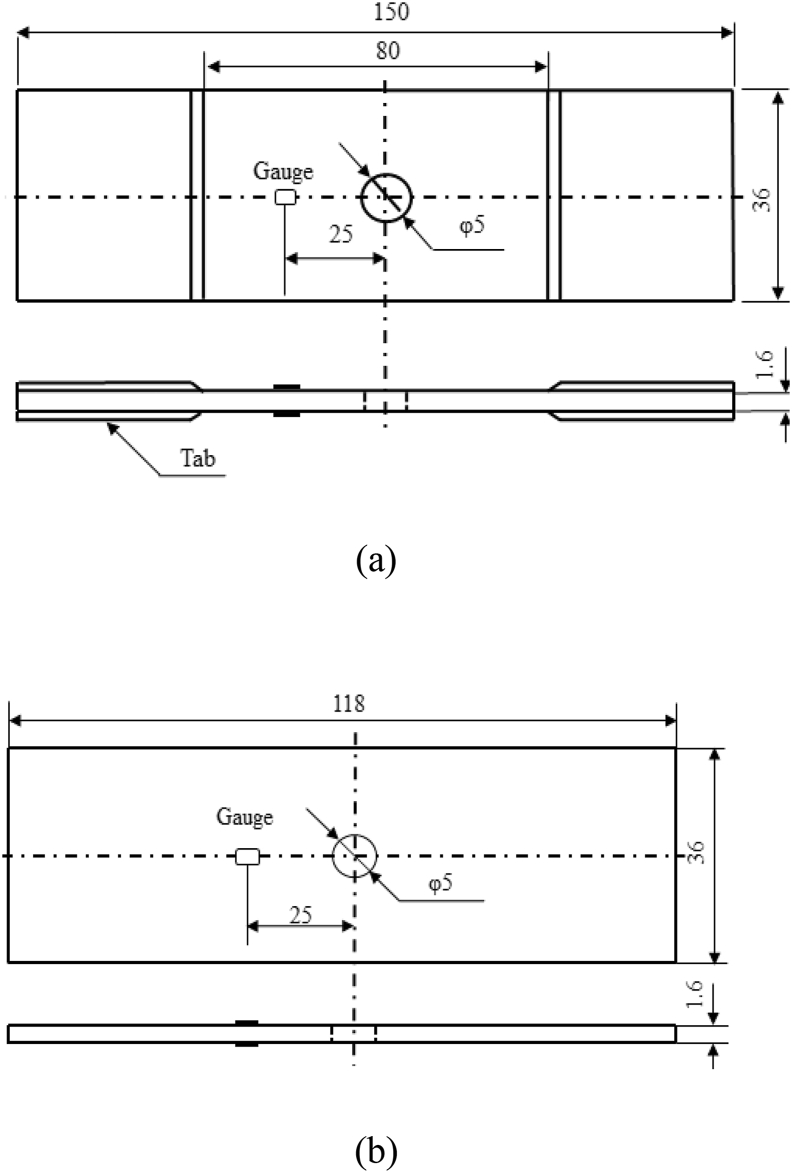
Specimen geometry. (a) Open-hole tension and open-hole tensile–tensile fatigue test specimen; (b) Open-hole compression test specimen.

The open-hole tensile test specimen has a length of 150 mm and a width of 36 mm in accordance with JIS K 7094, while the compression test specimen has a length of 118 mm and a width of 36 mm in accordance with JIS K 7093. A hole with a nominal diameter of 5 mm was made at the center of both specimens as shown in Figure 1(a), (b). Two kinds of machined holes were prepared: a drilled hole and a punched hole. Two strain gauges were attached 25 mm away from the center of the hole on the front and back faces of the specimen, and their average value was taken as the applied strain. For the tensile test specimen, tabs were attached at both ends of the specimen. The tabs made from the laminate with a length of 35 mm and a width of 36 mm were bonded to the specimen using an epoxy resin (105/206, West System), which was cured at 80 °C for 3 h. The geometry of the open-hole fatigue test specimen was identical to that of the open-hole tensile test specimen.

### 2.3 Hole making

#### 2.3.1 Drilling

The drilled hole was achieved using a drill bit for metals (SET50, NACHI) with 5 mm diameter, and was set as a baseline for comparison. The CFRP laminate was placed between the acrylic plates and it was drilled simultaneously to prevent peeling of the first and final layer of the CFRP laminate [21]. The traditional drilling process took a few seconds.

#### 2.3.2 Punching

**Figure 2:**
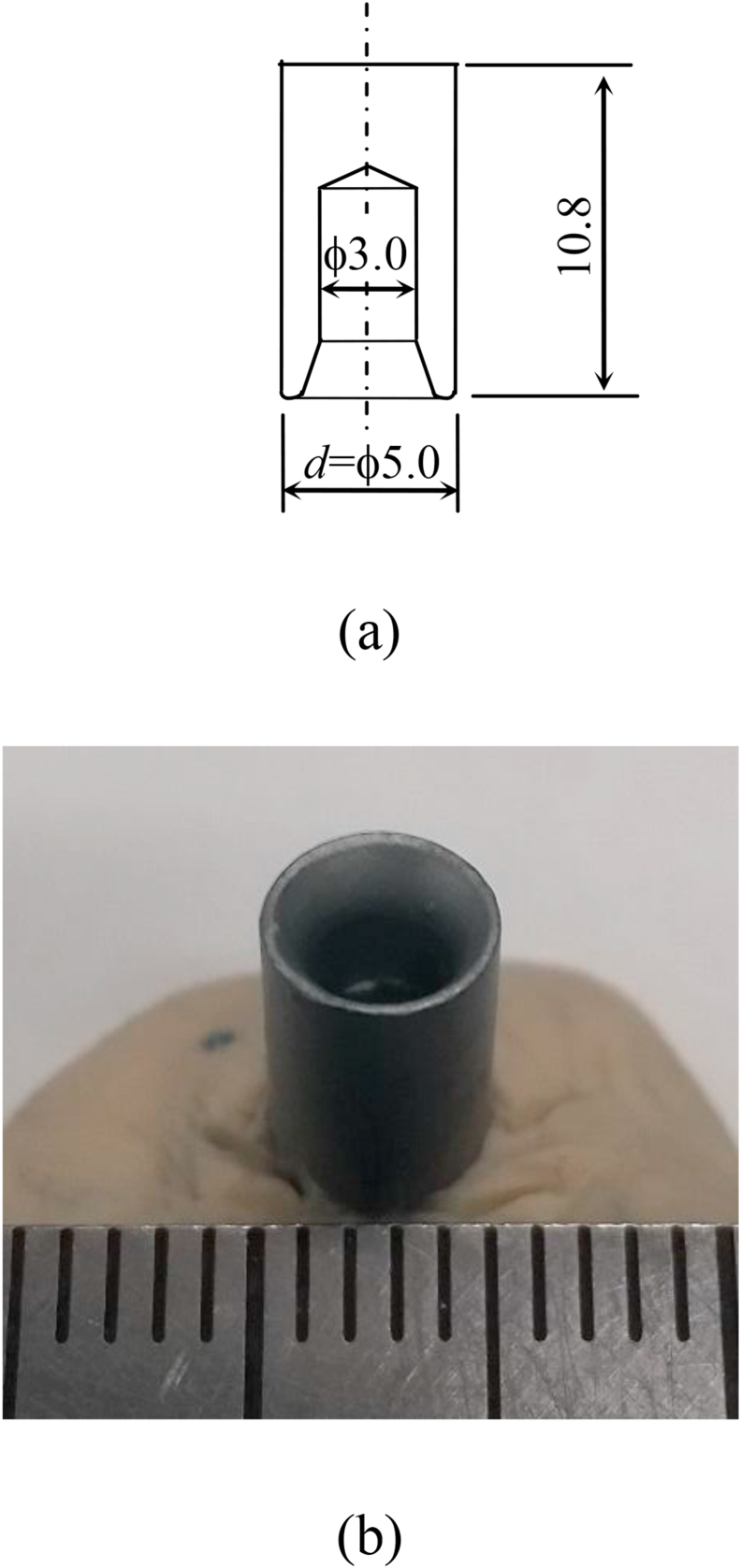
Punch tool. (a) Dimension; (b) The cutting tip.

The punch tool was originally developed (Fukui Byora Co., Ltd, Japan) in this study and the dimension is shown in Figure 2(a). The cutting tip of the punch tool is shown in Figure 2(b). The punch tool is a circular tube, and its outer diameter and length for this study were 5 mm and 10.8 mm, respectively. The punch tool was made of SCM435 using cold forging manufacturing.

**Figure 3:**
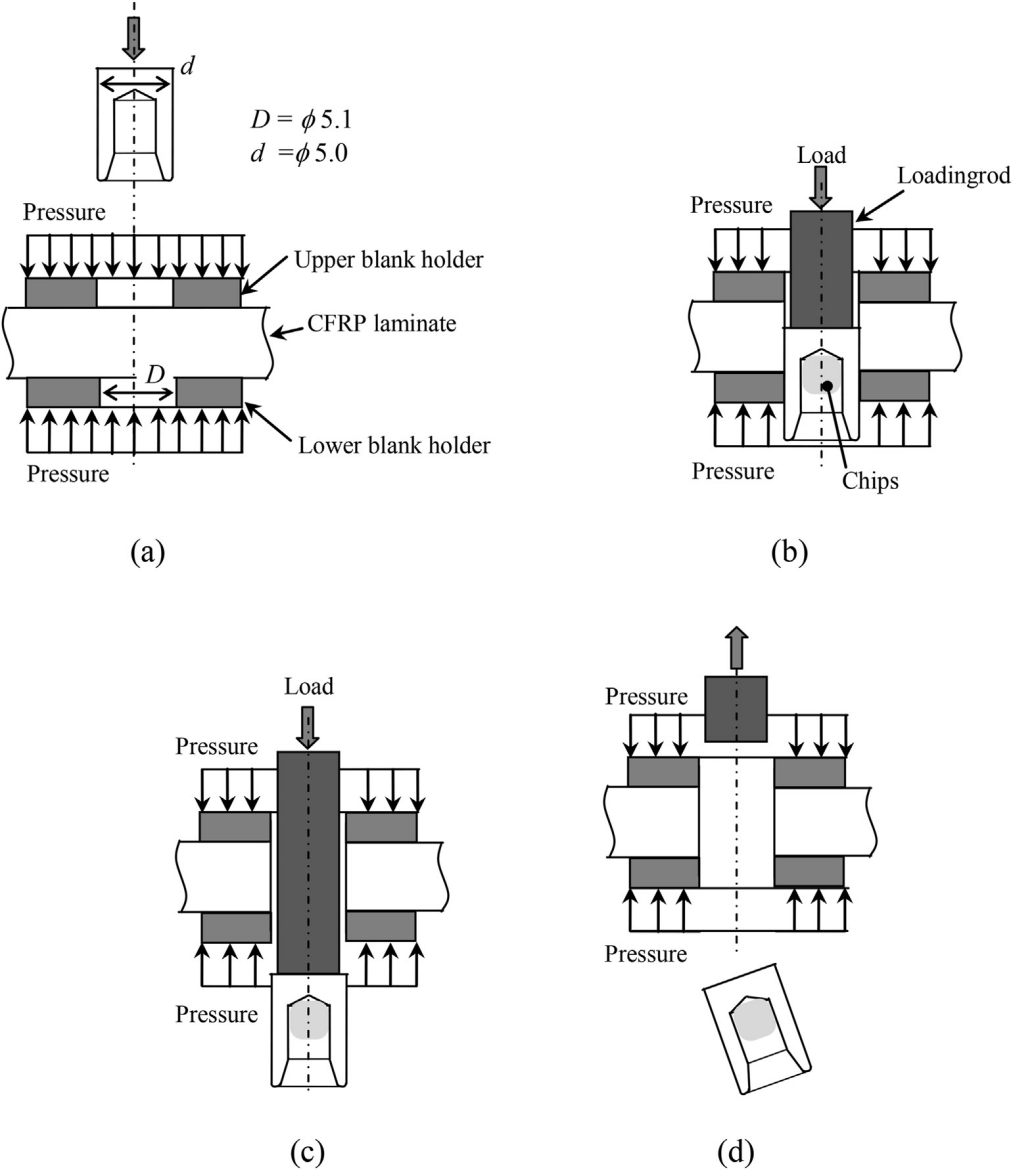
Punching process. (a) adjusting; (b) punching; (c) exit of the tool; (d) removal of loading rod.

The punching process of a CFRP laminate by the punch tool is depicted in Figure 3, which shows where the upper and lower blank holders, the CFRP laminate, and the punch tool were positioned. The upper and lower blank holders were pressed against the laminate and pressure was applied very close to the punching hole to prevent delamination (Figure 3a). To maintain the pressure, the punch tool was pressed into the laminate using a loading rod to make a hole in the laminate (Figure 3b). The chips that were cut from the laminate by the punch tool were removed through the lower blank holder together with the punch tool to complete the hole-making process (Figure 3c). Finally, the loading rod was returned to the original position (Figure 3d). The punching was performed using a riveting machine under control of displacement, which was the same used in the self-piercing riveting [22]. The punching process was performed within 1 s after positioning the laminates and punch tool.

The punch tool was replaced after each punching process, and as such a new punch tool was always used to make a punched hole. However, although the punch tool was not durable for repeated use, it can be produced cheaply and recycled using a cold forging manufacturing process.

The clearance between the hole diameters of the upper and lower blank holder as well as the clearance between the hole diameter of the blank holder and the diameter of the punch tool affected the quality of the punched hole. In this study, the hole diameters (5.1 mm) were the same for the upper and lower blank holders and the clearance between the diameters of the punch tool and the blank holder when the centers of the tool and the hole coincided was 0.05 mm. Smaller clearance could result in reduced damage to the laminate, although it was difficult to further reduce the clearance because of buckling of the punch tool due to increased punching load.

**Figure 4:**
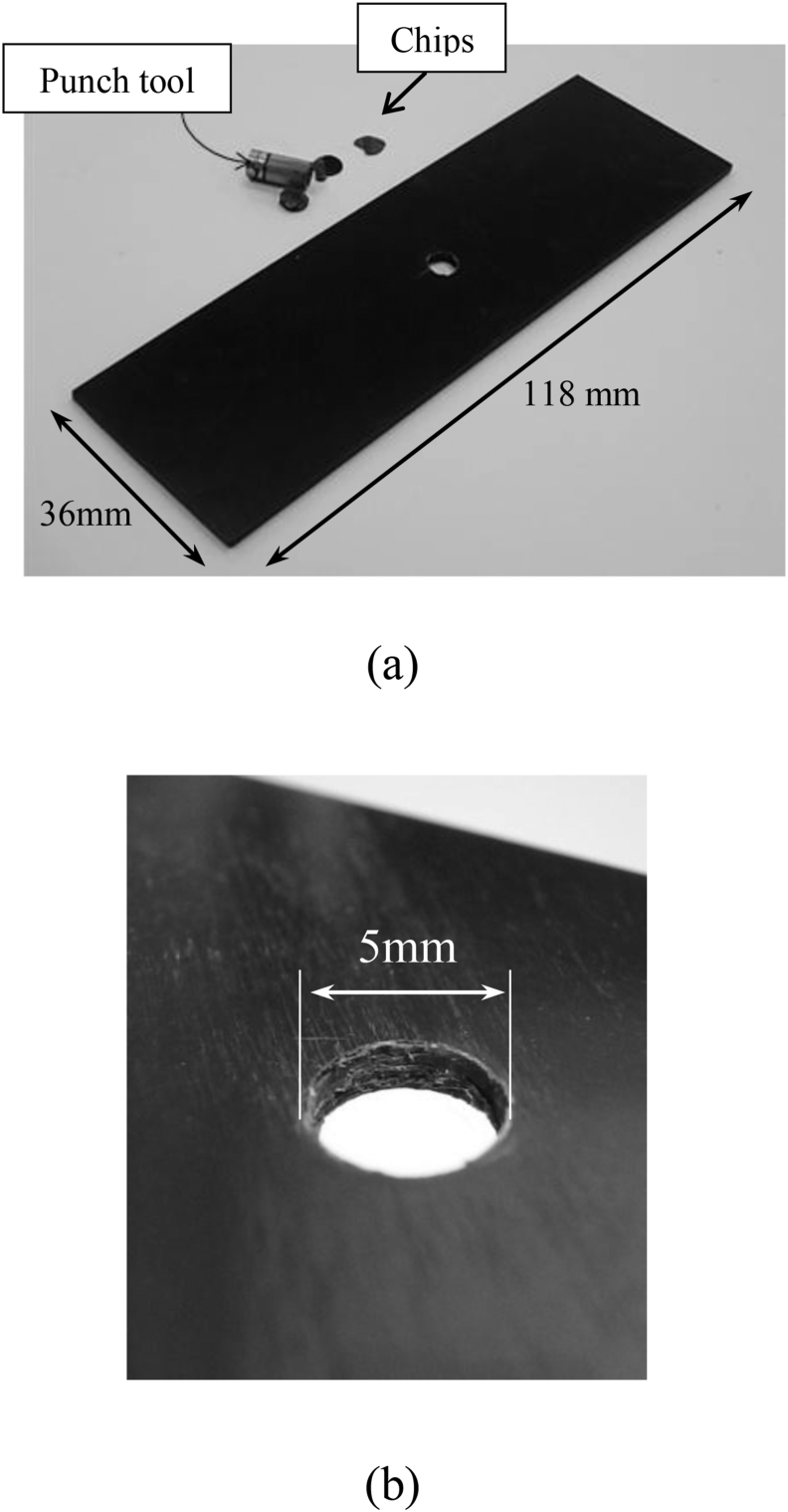
Punched hole of a CFRP laminate. (a) panoramic view; (b) closeup of the hole.

**Figure 5:**
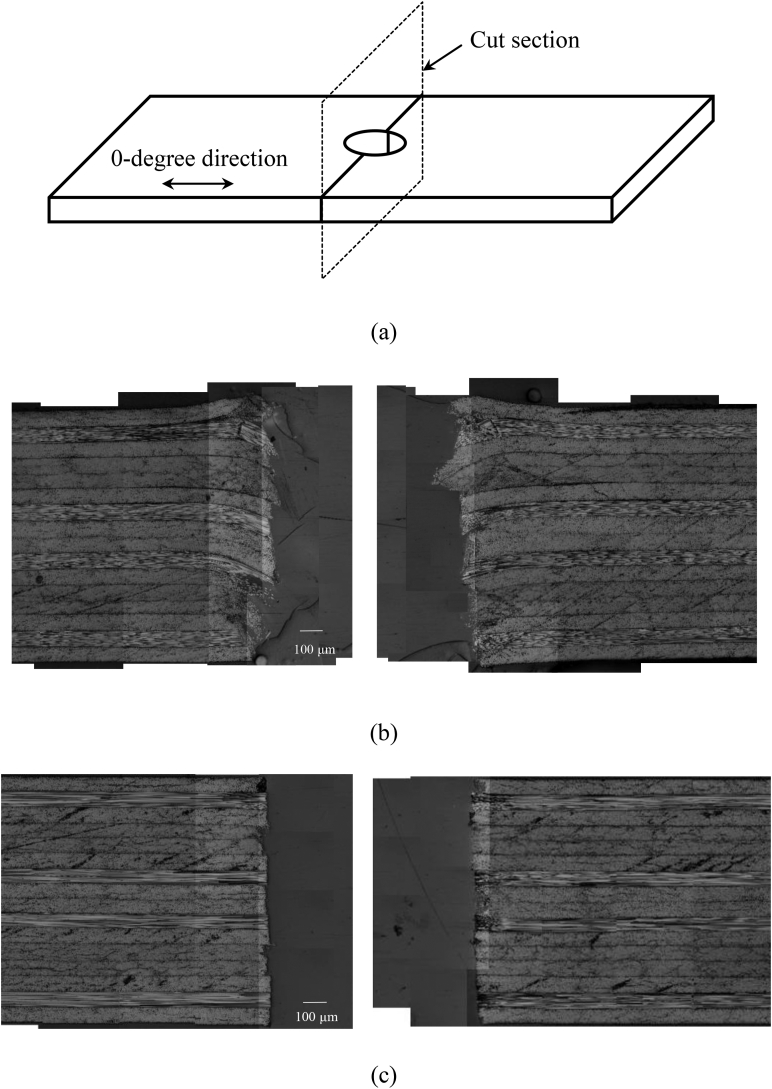
Cross-sections of the CFRP laminates at the hole section. (a) Cut section; (b) punched hole; (c) drilled hole.

Figure 4 shows a quasi-isotropic CFRP laminate with a punched hole. There is no visible damage such as cracks or splitting. Cross-sectional images of the punched hole and drilled hole are shown in Figure 5(b) and (c). An uneven surface around the punched hole as compared with that of the drilled hole was observed. Dragging of the plies was observed around the hole due to the shear loading during the punching process, and the average diameters of the punched hole and the drilled hole were 4.76 mm (cv. 0.7%) and 4.96 mm (cv. 0.8%), respectively. The diameter of the punched hole was smaller than the diameter of the punch tool due to the elastic recovery of dragged CFRP plies.

**Figure 6:**
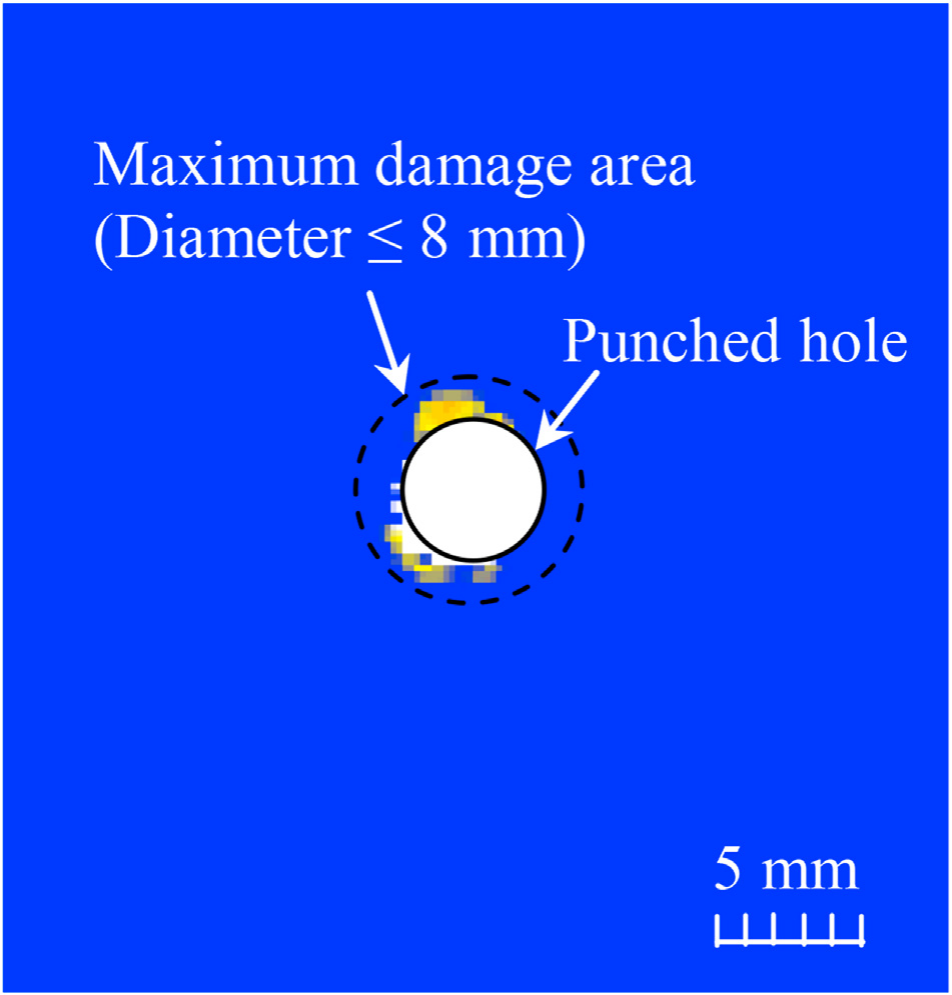
C-scan images of CFRP laminates after punching.

The delamination around the punched hole in the CFRP laminate was measured using ultrasonic inspection (SDS-5400R, Krautkramer). Figure 6 shows a C-scan image of the delamination. The maximum delamination area was approximately 1.5 mm around the punched hole.

### 2.4 Mechanical test procedure

#### 2.4.1 Open-hole tensile test

The open-hole tensile test was conducted using a universal testing machine (AG-IS 150kN, Shimadzu Corp.), performed at a testing speed of 1.0 mm/min. Five tensile test specimens were prepared in each hole-making process, resulting in a total of ten samples to be tested.

#### 2.4.2 Open-hole compression test

The open-hole compression test was performed using the universal testing machine, performed at a testing speed of 1.0 mm/min. An end-loading fixture (JIS K 7093) was used, and five compression test specimens were prepared in each hole-making process, resulting in a total of ten samples to be tested.

#### 2.4.3 Open-hole tensile–tensile fatigue test

A hydraulic fatigue testing machine (EHF-EG100kN-20L, Shimadzu Corp.) was employed for the open-hole tensile–tensile fatigue test to be conducted, at a stress ratio of *R* = 0.1 and a frequency of 5 Hz. The value of the frequency was selected to avoid heat generation while testing. The maximum stress varied from 45% to 90% of the OHT strength to obtain the relationship between a maximum stress and the number of cycles to failure (S–N curve). The sine wave motion was given at a rated load level, and more than 15 fatigue test specimens were prepared for each of the hole-making processes, resulting in more than 30 samples for testing.

## 3 Results

### 3.1 Open-hole tensile strength

**Figure 7:**
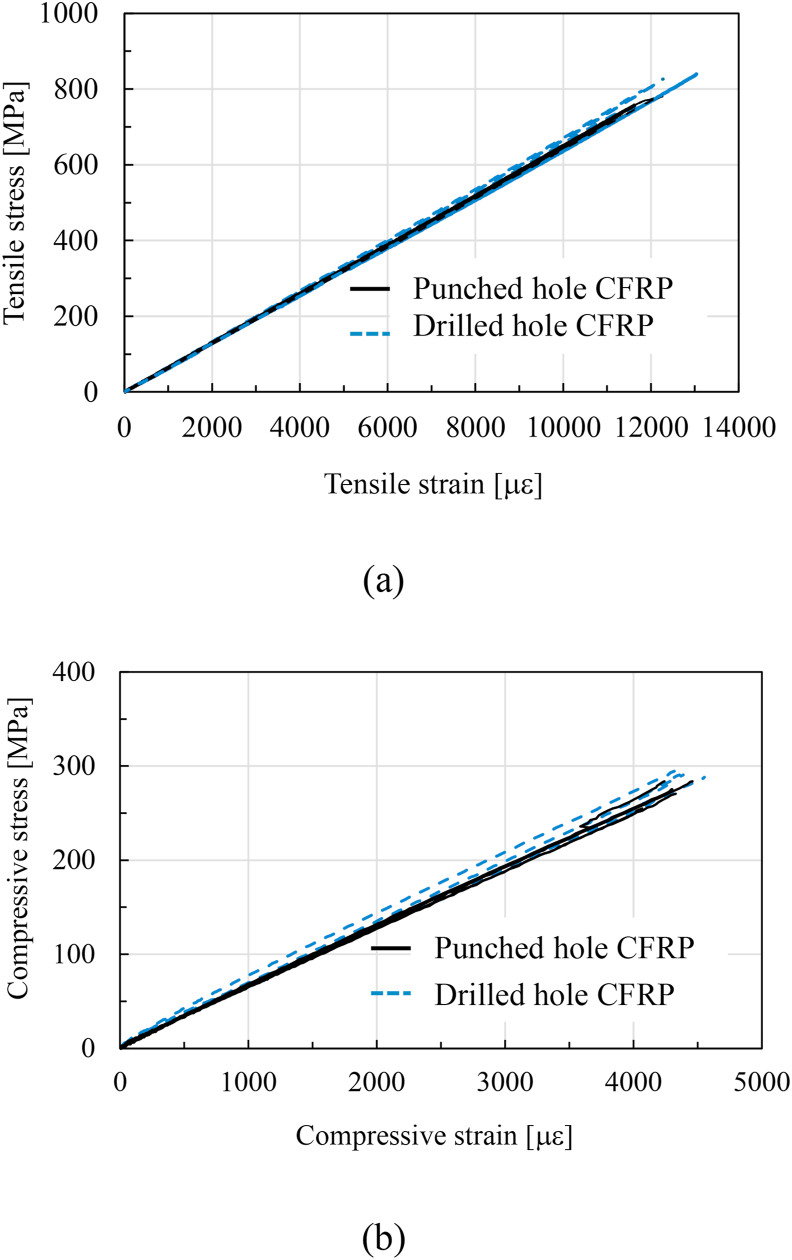
Stress–strain diagram of drilled- and punched-hole specimens. (a) Open-hole tensile test; (b) Open-hole compression test.

The tensile stress–strain diagram and the average OHT strength obtained from the tensile tests are presented in Figure 7(a) and summarized in Table 1, respectively. The tensile stress linearly increased until failure, with a corresponding increase in strain. All the specimens failed catastrophically by pull-out and delamination [23]. The OHT strengths of the drilled- and punched-hole specimens were comparable. The OHT strength of punched-hole specimens might be slightly larger than that of drilled-hole specimens because of the smaller hole diameter of punched hole. The reduction of the OHT strength and difference in the failure mode due to the uneven surface of the punched hole was not observed.

**Table 1:** Open-hole tensile and compressive strengths.

	OHT strength	OHC strength
Drilled hole specimens	766 ± 27 MPa	285 ± 10 MPa
Punched hole specimens	806 ± 30 MPa	273 ± 11 MPa

### 3.2 Open-hole compression strength

**Figure 8:**
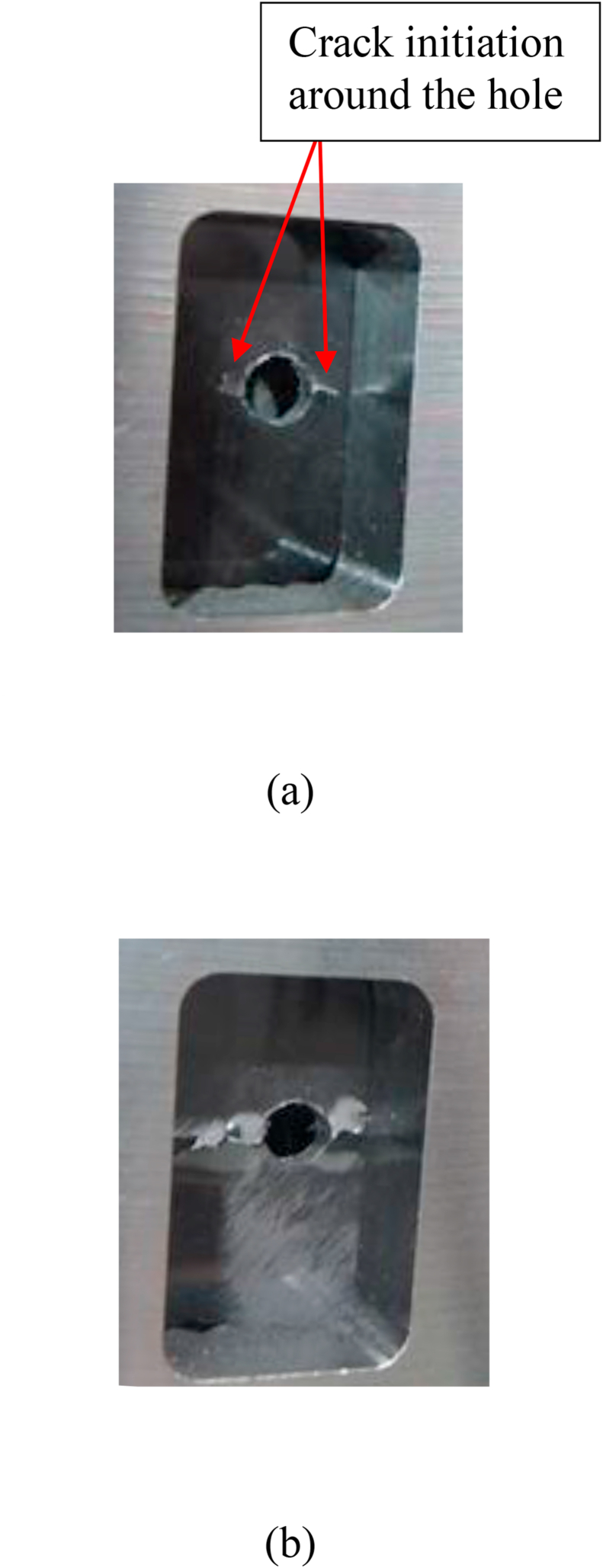
Compressive failure process. (a) Crack initiation; (b) final failure.

The compressive stress–strain diagram and the average OHC strength obtained from the compression tests are presented in Figure 7(b) and summarized in Table 1, respectively. The compressive stress linearly increased until failure occurred with a corresponding increase in the strains. A crack initiated in the vicinity of the hole (Figure 8a) and propagated horizontally (Figure 8b) by brittle failure [23]. There were no significant differences in the strength and failure modes between the drilled- and punched-hole specimens.

### 3.3 Open-hole tensile–tensile fatigue strength

**Figure 9:**
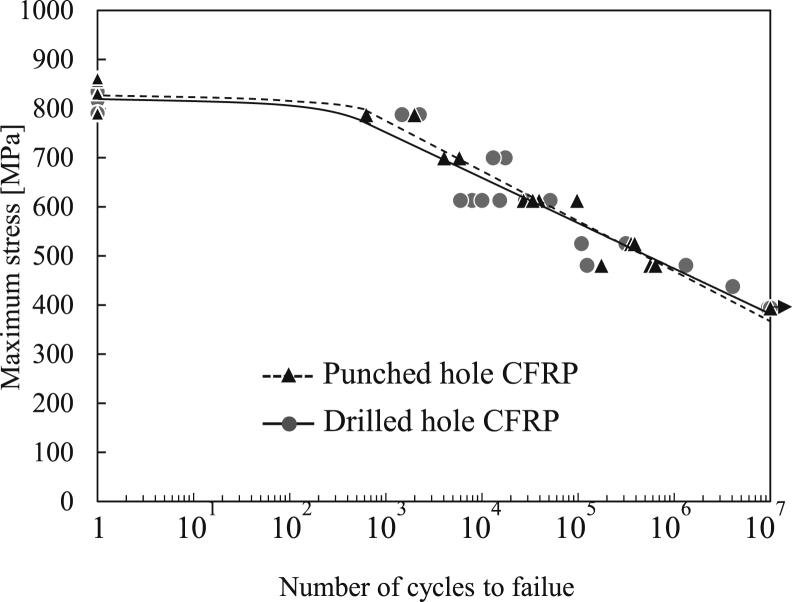
S–N curve by the fatigue tests of drilled- and punched-hole specimens.

The S–N diagram obtained from the tensile–tensile fatigue tests is shown in Figure 9. As the maximum stress decreased, the fatigue life for both the drilled- and punched-hole specimen increased. The S–N curve of the punched-hole specimen almost coincided with that of the drilled-hole specimen. Both the drilled- and punched-hole specimens did not fail at 10^7^ cycles, which corresponds to 45% of their OHT strengths. This value is defined as the fatigue limit of the specimens. The punching process did not alter the fatigue limit and lifetime of the specimen since the S–N diagram did not show distinct variation between the drilled- and punched-hole specimens.

## 4 Discussion

### 4.1 The effect of surface quality on the mechanical property

The comparison between the two hole-making processes did not show significant differences in the mechanical property of the punched-hole CFRP laminates. It was also reported that the effect of conventional machining induced surface damage on crack initiation and propagation in CFRP laminates was not distinctive [24]. However, the hole shapes affect the compressive failure of CFRP laminates [25]. An extremely smooth surface finish on the drilled hole improves the fatigue strengths of CFRP laminates [21]. Although the uneven surface of the drilled hole was observed in this study, the optimized drilling process parameters and drill tip geometry further improve the surface quality of a hole [26, 27, 28, 29, 30]. The effect of machining induced damage on microscopic crack initiation and propagation still needs to be studied [31].

### 4.2 Application for dissimilar materials; a CFRP laminate and a mild steel plate

**Figure 10:**
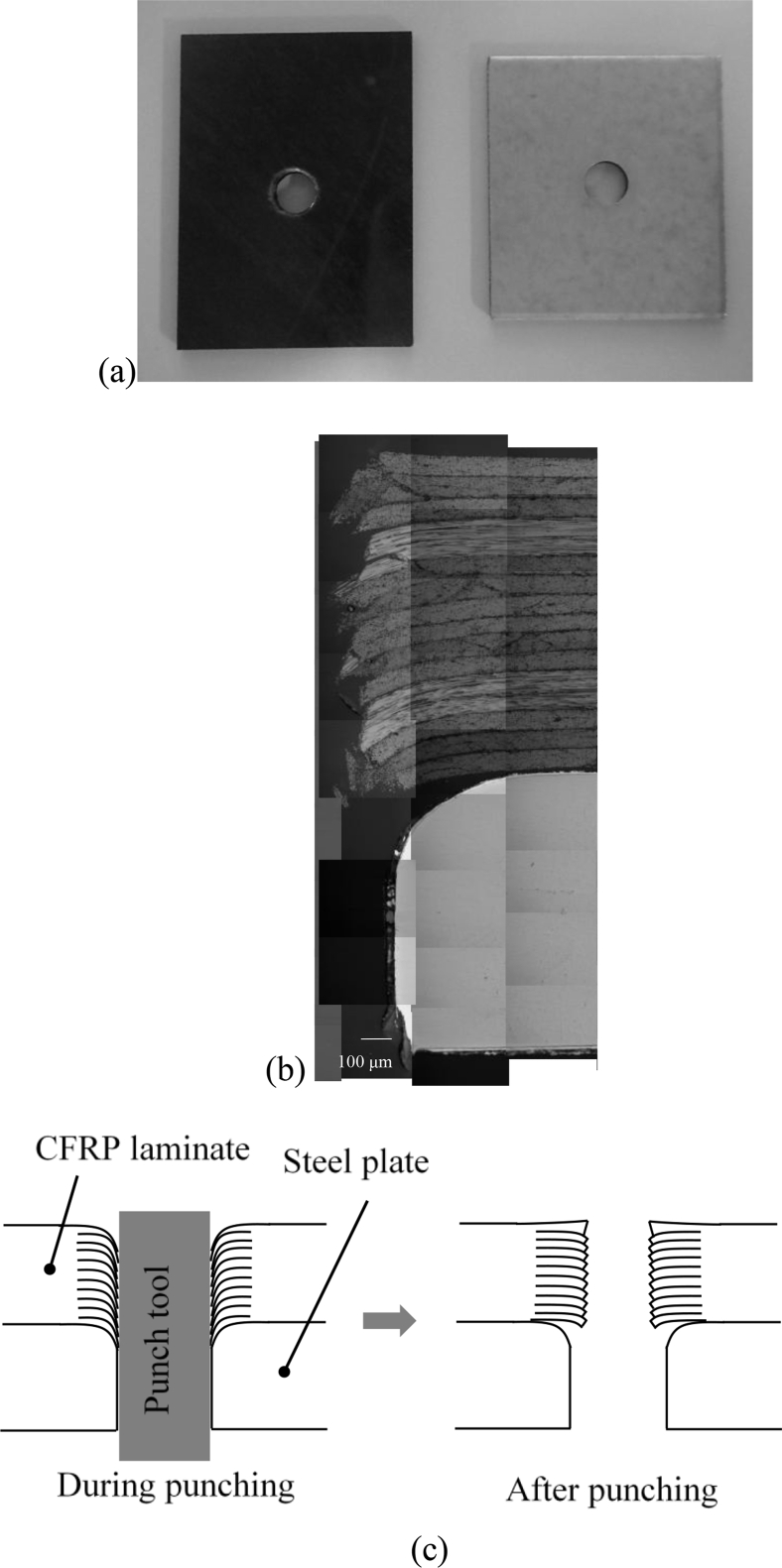
Hole making for dissimilar materials punched from CFRP to steel plate. (a) Macroscopic observation; (b) the cross-sectional observation; (c) schematic image.

A CFRP laminate and a mild steel plate punched simultaneously using the 5 mm diameter punch tool are shown in Figure 10(a). The punch tool pierced from the CFRP laminate to the mild steel plate. Visible damages such as crack or splitting were not observed on the surfaces of the CFRP laminate.

Figure 10(b) shows the cross-sectional observation of the punched hole. The uneven surface was observed for the CFRP laminate while the smooth surface was for the mild steel plate. The diameter of the hole was smaller for the CFRP laminate than the mild steel plate. CFRP plies were dragged by the punch tool due to the shear force during the punching process. The dragged plies elastically recover after punching, and therefore, the hole diameter of the CFRP laminate became smaller than the diameter of the punch tool (Figure 10c). The punching technique can be applied in the hole-making process for CFRP laminates and metal plates, although some differences in diameter could develop.

## 5 Conclusions

Tensile, compressive, and tensile–tensile fatigue tests were performed on quasi-isotropic CFRP laminates (*V*_*f*_ = 65%) with an inserted hole made by punching and drilling. The applicability of the punching technique as a highly productive hole-making process for structural CFRP laminate was investigated in terms of the mechanical properties. The results obtained in this study were summarized as follows.

(1) The same hole diameter for upper and lower blank holders and a small clearance of 0.05 mm between the diameters of the punch tool and blank holder were selected to suppress damages during the punching process. There were no visible cracks or splitting observed macroscopically. The surface roughness of the punched hole, however, was still uneven as compared with that of the drilled hole due to the dragging of the CFRP plies during punching, and the delaminated area was found to be less than 1.5 mm for the 5 mm nominal punched-hole diameter.
(2) The open-hole tensile, compressive, and tensile–tensile fatigue strengths of the CFRP laminate with the punched hole were comparable to those with the drilled hole. The damages caused during the punching or drilling process had no significant effect on the variations in the mechanical properties of the laminate.
(3) It was shown that a CFRP laminate and a mild steel plate could be punched simultaneously without visible crack or splitting. However, there was some difference in diameter where the hole diameter in the CFRP laminate was smaller than that in the mild steel plate due to the elastic recovery of the dragged CFRP plies.

## Declarations

### Author contribution statement

Masahito Ueda: Conceived and designed the experiments; Wrote the paper.

Vu Manh Cuong: Performed the experiments; Analyzed and interpreted the data; Contributed reagents, materials, analysis tools or data.

Atsushi Yamanaka: Performed the experiments; Analyzed and interpreted the data.

Kazuhiro Sakata: Conceived and designed the experiments.

### Funding statement

This research did not receive any specific grant from funding agencies in the public, commercial, or not-for-profit sectors.

### Data availability statement

Data will be made available on request.

### Declaration of interests statement

The authors declare no conflict of interest.

### Additional information

No additional information is available for this paper.
